# Ultrahigh-resolution imaging of water networks by atomic force microscopy

**DOI:** 10.1038/ncomms14313

**Published:** 2017-02-03

**Authors:** Akitoshi Shiotari, Yoshiaki Sugimoto

**Affiliations:** 1 Department of Advanced Materials Science, The University of Tokyo, Kashiwanoha 5-1-5, Kashiwa 277-8561, Japan

## Abstract

Local defects in water layers growing on metal surfaces have a key influence on the wetting process at the surfaces; however, such minor structures are undetectable by macroscopic methods. Here, we demonstrate ultrahigh-resolution imaging of single water layers on a copper(110) surface by using non-contact atomic force microscopy (AFM) with molecular functionalized tips at 4.8 K. AFM with a probe tip terminated by carbon monoxide predominantly images oxygen atoms, whereas the contribution of hydrogen atoms is modest. Oxygen skeletons in the AFM images reveal that the water networks containing local defects and edges are composed of pentagonal and hexagonal rings. The results reinforce the applicability of AFM to characterize atomic structures of weakly bonded molecular assemblies.

## Abstract (web summary)

The structure of water in the first layer on surfaces is essential to our understanding of various phenomena, such as surface wettability and heterogeneous catalysis. Here, the authors use atomic force microscopy with a CO-functionalized tip to image water defects on copper surface at atomic resolution.

Mechanisms of ice growth on metal surfaces are determined by the adsorption structure of the first water layer. Water in the first layer on the surfaces creates various hydrogen (H)-bonding networks, including one-, two- and three-dimensional (1D, 2D and 3D) structures, where the ice rules^1^ are frequently broken. The water networks on well-ordered metal surfaces generally form periodic phases, which readily turn into other phases depending on the surface structures, temperature and coverage. Moreover, heterogeneous catalysis of metals facilitates dissociative reactions of water to yield hydroxyl (OH) groups, which further complicates the structures of the H_2_O–OH mixed layers. The configurations of the first water layers have been investigated with spectroscopic methods, such as vibrational spectroscopy, photoelectron spectroscopy and thermal desorption spectroscopy^2^,^3^,^4^. Although these methods are well-established and easily analysable, they average out wide-range information on the surface. Therefore, local structures (vacancies, impurities, dissociative products and layer edges), which may have different configurations and properties from the intact water networks, are generally undetectable or buried in the main peaks in the spatially averaged spectra. However, such local defects in water layers on metals play an important role in the catalysis and wettability; on copper (Cu) surfaces, for example, OH groups initiate a low-temperature water-gas shift reaction^5^, and behave as anchors for adsorbing onto the substrate^6^ and as bonding sites for the second water layer^7^.

Scanning tunnelling microscopy (STM) is known to be a useful tool for investigating local structures in water networks at the single-molecule level. Since various H-bonding networks on metal surfaces have already been observed directly and characterized, STM brings innovative information to the experimental methodology of water–solid interfaces^3^,^4^,^8^,^9^,^10^,^11^,^12^. Hitherto STM was employed to trace thermal diffusion of individual water molecules^13^, induce dissociation of water^14^,^15^,^16^, fabricate H-bonding complexes^16^,^17^,^18^ and control H-atom dynamics^16^,^17^,^18^,^19^,^20^,^21^ on surfaces. In particular, recent STM studies visualized the intramolecular structures of water monomers and small clusters on an ultrathin NaCl film^18^ and controlled the direction of H-bonds in the cluster^20^. On the other hand, it remains difficult to identify the atomic configurations of self-assembled water networks on metal surfaces by topographic STM images alone, and thus STM-based information is best supported with vibrational spectroscopy and/or theoretical calculations^7^,^22^,^23^. This is mainly because STM images reflect both electronic states near the Fermi level and geometries of the adsorbates.

As well as STM, non-contact atomic force microscopy (AFM) is a powerful option for visualization and analysis of surface structures^24^. Non-contact AFM has a remarkable range of applications, including atomic-resolution imaging of insulators^24^ and chemical identification of individual atoms^25^; however, water-layer studies using non-contact AFM have been notably scarce^26^. For aromatic molecules on surfaces, on the other hand, Gross *et al*.^27^ have proposed that the spatial resolution of AFM can be significantly enhanced by using probe tips functionalized by molecules such as carbon monoxide (CO). Carbon skeletons in the molecules can then be visualized^27^ owing to the sensitive detection of the repulsive forces between atoms in the adsorbate and the sharpened tip apex^28^. H bonds between organic molecules are also potentially visualized^29^, even though the results of these imagings remain controversial^28^,^30^. This imaging technique has already been applied to various organic molecules^31^,^32^ and non-carbon materials such as iron clusters^33^ and metal chalcogenide thin films^34^,^35^. In contrast to the rigid and stable materials, water molecules in the first layers would form various adsorption geometries differing from the monomeric configurations, owing to H bonds^16^,^18^,^22^,^36^. Therefore, it was unobvious whether AFM can/cannot non-destructively visualize atomic structures of the weakly bonded molecular assemblies.

Here, we aim to visualize water networks on a metal surface with non-contact AFM at much higher spatial resolution than in existing STM images. We focus on water adsorbed onto a Cu(110) surface, on which many different kinds of H-bonding networks can be formed^7^,^16^,^19^,^22^,^23^,^37^,^38^,^39^,^40^,^41^.We demonstrate the application of AFM with a functionalized probe tip to the visualization of water networks, including their defects, edges and domain boundaries.

## Results

### Visualization of 1D water chains on Cu(110)

**Figure 1:**
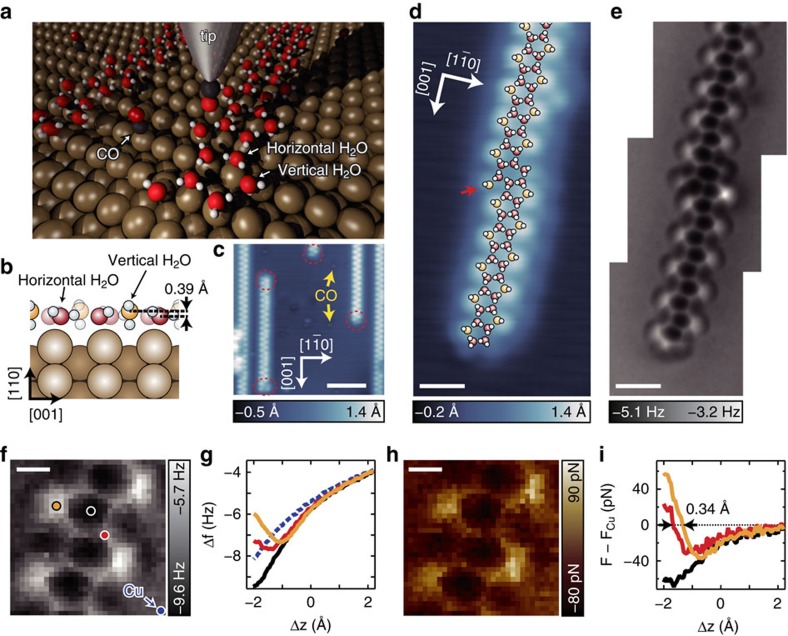
Visualization of pentagonal water chains and their defects. (**a**) Schematic of STM/AFM measurement for pentagonal water chains on Cu(110) with a CO-terminal tip. Red, black, white and brown spheres show O, C, H and Cu atoms, respectively. (**b**) Side-view schematic of the water chain^22^. Red (yellow) spheres represent O atoms of horizontal (vertical) H_2_O. (**c**) STM image of the water chains on Cu(110) with a CO-terminal tip (sample bias *V*=30 mV, tunnelling current *I*=20 pA). The zigzag chains have terminals (red ellipses). (**d**,**e**) STM (*V*=30 mV, *I*=20 pA) and AFM (*V*=0 mV, oscillation amplitude *A*=2 Å) images, respectively, of a water chain including a kink and a terminal. An atomic structure of the chain is superposed in **d**. Note that the other possible structure is shown in Supplementary Fig. 5. The tip height in **e** was set over the bare surface under the same conditions as in **d**. (**f**) Δ*f* map of the pentagonal chain at a tip height Δ*z*=−2 Å (*A*=1 Å). (**g**) Δ*f*(*Δz*) curves recorded over the markers in **f**. (**h**) Force map of the chain at Δ*z*=−1.95 Å after subtraction of the force for the bare surface *F*_Cu_. (**i**) Force curves over the makers in **f** after subtraction of *F*_Cu_(Δ*z*). Scale bars, 50 Å (**c**); 10 Å (**d**,**e**); 3 Å (**f**,**h**).

A clean Cu(110) surface was exposed to H_2_O gas at 78 K and then 1D water chains along the [001] direction are formed^22^,^38^,^39^,^40^,^41^. In a previous study using STM, vibrational spectroscopy and theoretical calculations^22^, these chains were proposed to consist of fused pentagonal rings (Fig. 1a,b). In the pentagonal unit, one H_2_O is bonded onto the trough between the atomic Cu rows along the 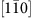 direction (near the hollow site) in a vertical orientation relative to the surface (vertical H_2_O; the yellow spheres in Fig. 1b) and the others lie on the Cu row (near the atop site) with almost flat configurations (horizontal H_2_O; the red spheres in Fig. 1b). In the STM image, the protrusions aligned along the chain direction in a zigzag manner are ascribed to vertical H_2_O (ref. 22).

Figure 1c shows typical STM image of the water chains on Cu(110) at 4.8 K. By using a probe tip functionalized by a CO molecule^27^,^42^ (Fig. 1a), the zigzag shape of the STM image is emphasized (Supplementary Fig. 1 and Supplementary Note 1); however, the pentagonal bonding structure is still hardly discriminated. The zigzag chains have ‘terminals' where the chain elongation along the [001] direction is ended (the red ellipses in Fig. 1c), and ‘defects' where the zigzag manner is locally broken. Some of the defects correspond to ‘kinks' deflecting the 1D chains (Fig. 1d). An AFM image of the water chain with the CO-terminal tip (Fig. 1e) clearly reflects the atomic position of O atoms, in contrast with an STM image of the same area (Fig. 1d). For AFM, the frequency shift (Δ*f*) was measured in constant-height mode^27^. An intact chain is observed as fused pentagonal rings in complete agreement with the model proposed previously (Fig. 1a,b). To identify the source of the observed structure, a 3D force map^43^ was recorded over the pentagonal chain (Fig. 1f–i; see also Supplementary Fig. 2 and Supplementary Note 2). The imaging mechanism is consistent with that for organic molecules; the Pauli repulsion between the atoms on the surface and CO at the tip apex leads to high Δ*f* contrasts^27^. The zero-force distance for vertical H_2_O (the yellow curve in Fig. 1i) is displaced by 0.34 Å towards the vacuum relative to that for horizontal H_2_O (red), because the O atom of vertical H_2_O is 0.39 Å more protruded than that of horizontal H_2_O (ref. 22), as shown in Fig. 1b. As a result of this displacement, vertical H_2_O is the most repulsive to the tip at a tip height Δ*z* of about −2 Å (Fig. 1h,i) and the Δ*f* intensity of vertical H_2_O at the tip height (the yellow curve in Fig. 1g) is higher than that of horizontal H_2_O (red). On the other hand, the centre of a pentagon where no molecules exist (the black dot in Fig. 1f) is still attractive at Δ*z*≈−2 Å (the black curve in Fig. 1i) due to attractive interactions of the surrounding molecules such as van der Waals forces.

Although the atomic structures of the pentagonal unit (Fig. 1b) is asymmetric with respect to the 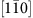 axis (because of the locations of H atoms; see Supplementary Fig. 3), the pentagonal ring in the AFM image in Fig. 1e appears almost symmetric. This implies that the AFM image shows the ‘apparent' bonds between adjacent O atoms, rather than the O–H covalent bonds and H bonds themselves, due to the relaxation (tilting) of CO at the tip apex^28^,^30^. The presumption is reinforced by our simulations of AFM images using the Probe Particle Model provided by Hapala *et al*.^28^ (Supplementary Fig. 4 and Supplementary Note 3). Moreover, H atoms (in particular, the dangling H atom of vertical H_2_O) may be displaced slightly away from the tip approached^44^, which also account for the absence of any significant effect of H atoms on the image. Imaging with a tip terminated by another atom/molecule would be helpful to identify the H-atom positions; the literature shows that the direction of H bonds in a water cluster can be resolved and manipulated by using STM with a Cl-terminated tip^18^. Nevertheless, the AFM image provides much valuable information about the local defects. Based on the image in Fig. 1e, the atomic structure of the chain terminal and kink can be determined as schematically shown in Fig. 1d. The terminal retains its pentagonal ring. The kink is ascribed to a hexagonal ring with an additional vertical H_2_O (the red arrow in Fig. 1d). Note that Fig. 1d shows a possible structures of the kink and that the other orientation of horizontal H_2_O in the hexagonal ring is conceivable (see Supplementary Fig. 5 and Supplementary Note 4).

### Characterization of local defects in 1D water chains

**Figure 2:**
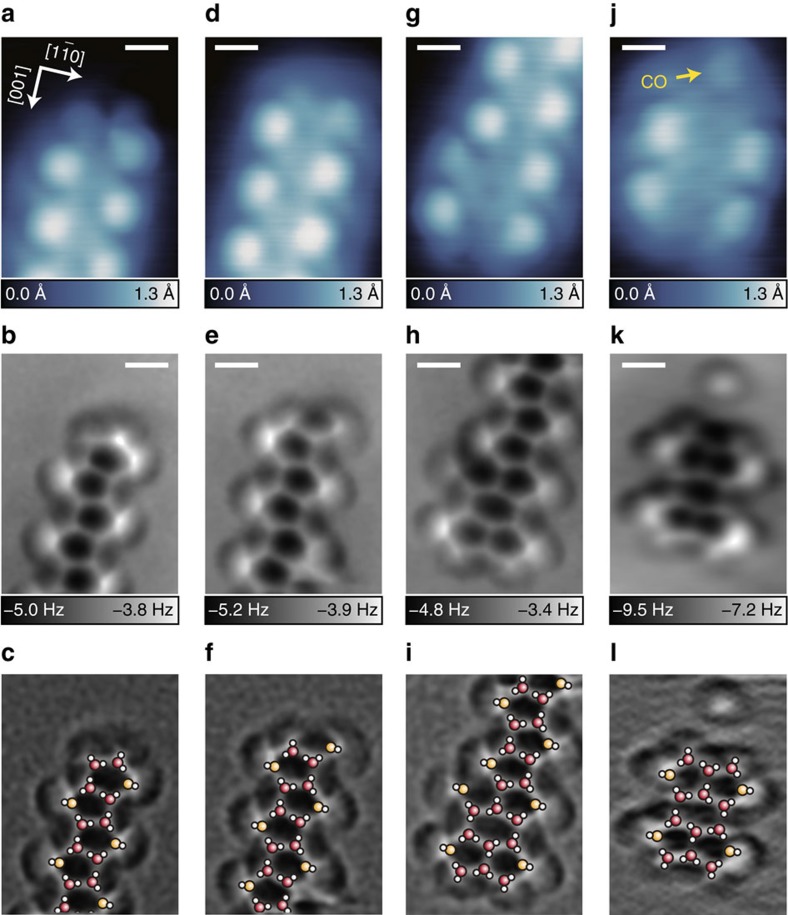
High-resolution images of terminals in water chains. STM (top) and AFM (middle) images of terminals for the pentagonal water chains. An atomic structure of each terminal is superposed on the Laplacian-filtered AFM image (bottom). (**a**–**c**) Pentagonal terminal. (**d**–**f**) Pentagonal terminal with an additional vertical H_2_O. (**g**–**i**) Fused hexagonal and pentagonal terminal. (**j**–**l**) ‘Tetraphyllous cluster' consisting of four pentagons. The images in **a**,**b** are magnified and 180°-rotated versions of those in Fig. 1d,e, respectively. The images in **d**,**g** were obtained at *V*=30 mV and *I*=20 pA, the image in **j** at *V*=50 mV and *I*=20 pA, the images in **e**,**h** at *V*=0 mV, *A*=2 Å, and Δ*z*=0 Å, and the image in **k** at *V*=0 mV, *A*=1 Å, and Δ*z*=0 Å. The tip height Δ*z* in **e**,**h**,**k** was set over the bare surface under the same conditions as in **d**,**g**,**j**, respectively. Scale bar, 5 Å.

The high-resolution AFM images enable each local defect in the 1D water chains to be characterized. Three kinds of terminals are mainly observed: a pentagonal ring (type i; Fig. 2a–c), a pentagonal ring with an additional vertical H_2_O (type ii; Fig. 2d–f) and two fused pentagonal rings (type iii; Fig. 2g–i). The STM image of type i (Fig. 2a) is very similar to that of type ii (Fig. 2d), but the corresponding AFM images clarify the structural difference (Fig. 2b,e). We also find a small cluster imaged as tetraphyllous-shaped protrusions with STM (Fig. 2j). The corresponding AFM image (Fig. 2k) indicates that the cluster is composed of a hexagonal ring surrounded by four pentagonal rings, suggesting that it consists of 16 H_2_O molecules (Fig. 2l). Because the cluster is predominantly observed at relatively low coverages at 78 K (Supplementary Fig. 6), this is a stable structure at the initial stage. This cluster has a similar structure to the terminal of type iii; at the terminal, the 1D chain is elongated from one of the four fused pentagonal rings, whereas the nearest neighbouring pentagonal ring is broken (Fig. 2i). This suggests that the ‘tetraphyllous cluster' probably corresponds to a core for growing the 1D chain to yield a type-iii terminal. We also observed several kinds of defects and kinks, which are composed of fused pentagonal (and hexagonal) rings (Supplementary Fig. 7). Note that OH groups are probably included in some of the fused hexagonal defects (Supplementary Fig. 8 and Supplementary Note 5).

### High-resolution imaging of a water-hydroxyl network on Cu(110)

**Figure 3:**
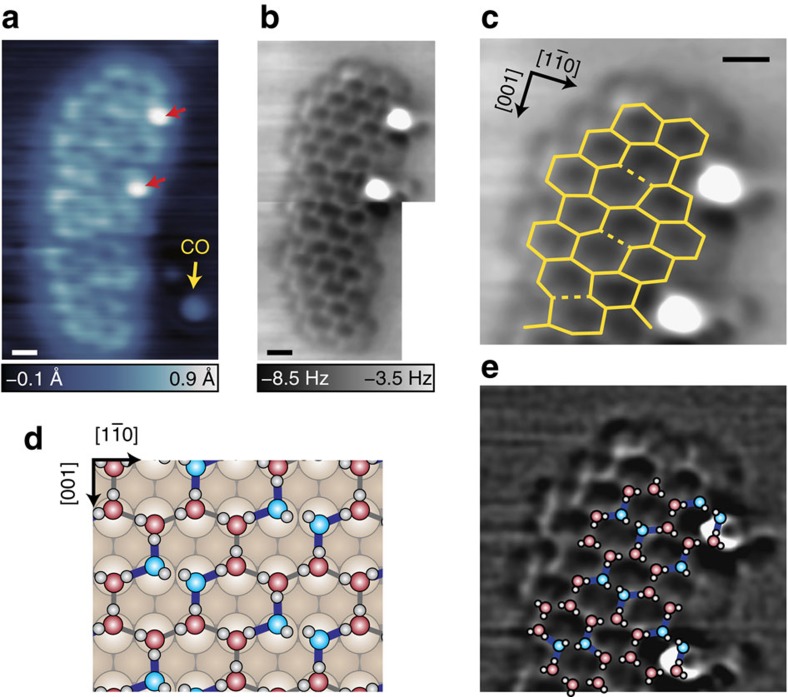
Visualization of a hexagonal water–hydroxyl island. (**a**) STM image of H_2_O/Cu(110) after annealing at 160 K (*V*=50 mV, *I*=20 pA). The image was obtained at 4.8 K with a CO-terminal tip. (**b**) AFM image of the island in **a** (*V*=0 mV, *A*=1 Å, Δ*z*=−1 Å). The tip height Δ*z* was set over the bare surface under the same conditions as **a**. (**c**) AFM image magnified at the upper part of **b**. The solid (dotted) lines represent the apparent O–O bonds with lengths of less (more) than 3.1 Å. (**d**) Schematic structure of the *p*(2 × 6) H_2_O–OH network on the surface^7^. (**e**) Proposed inside structure of the island superposed on the Laplacian-filtered image in **c**. Note that other possible structures are shown in Supplementary Fig. 5. Scale bar, 5 Å.

Even if H atoms are almost invisible, the distance between O atoms correlates highly with the strength of H bonds and can be sensitively detected by AFM with CO-terminal tips. To demonstrate that, we observed a H_2_O–OH mixed network on Cu(110), which was formed by partial dissociation of water after the sample was annealed at 160 K. At the temperature, OH groups are yielded by water dissociation and coexists with remaining H_2_O molecules^37^. The islands appear as ‘crooked bands' elongated along the [001] direction (Fig. 3a; see also Supplementary Fig. 9 and Supplementary Note 6), which are quite similar to the appearance of H_2_O/O/Cu(110) at 155 K (ref. 41). However, the atomic structure of the bands was not resolved by STM (ref. 41). On the other hand, a CO-terminal tip enables the STM image to show a honeycomb structure (Fig. 3a). In the AFM image at the same region, each O atom is resolved (Fig. 3b). This island is composed only of hexagonal rings, whereas we also find several pentagonal rings remaining at the edges of another island (Supplementary Fig. 9). Remarkably, the hexagonal rings (especially inside the island) in Fig. 3b seem to be asymmetric. The superposed lines in Fig. 3c show the apparent O–O bonds in the oxygen skeleton of the island. In the image, three O–O bonds (dotted lines; with the lengths of ∼4 Å) are much longer than the other bonds (solid lines; 2–3 Å). The inhomogeneous O–O distances originate from the mixture of intact H_2_O molecules and dissociative OH groups.

We ascribe this structure to the *p*(2 × 6) network with a component ratio of 2 H_2_O:1 OH (refs 7, 45; see Fig. 3d). In the network, two OH groups face each other without H-bonds, namely, Bjerrum defects^7^. These defects have no H bond between the OH groups, whereas OH behaves as a good acceptor to provide strong H bonds with the two adjacent H_2_O molecules (blue lines in Fig. 3d). Therefore, the hexagonal ring shows a long O–O side between the OH groups (at a distance of 3.2 Å), two short sides between H_2_O and OH (2.5 Å) and four middle-level sides between H_2_O molecules (2.7–2.8 Å). We assign the long O–O bonds (the dotted lines in Fig. 3c) to Bjerrum defects. The direction of the long O–O bonds is partially oriented, corresponding to a domain boundary. We tentatively proposed the inside structure of the island including the boundary as shown in Fig. 3e. This assignment indicates that the apparent O–O bond length between the OH groups is 4.0±0.2 Å, that between H_2_O and OH is 2.5±0.3 Å and that between H_2_O molecules is 2.8±0.2 Å (Supplementary Fig. 10 and Supplementary Note 6). Although the apparent bond lengths in AFM images with CO-terminal tips become exaggerated^46^, the bond distances are in good agreement with those in the theoretical model (Fig. 3d). According to the model, O atoms of both H_2_O and OH locate at an almost identical height along the surface normal^45^, which is also in good agreement with the similar appearance of the hexagonal vertices in the AFM image. The experimental image is comparable to our simulated AFM images based on the model in Fig. 3d, rather than those for the ‘H-down' model consisting of H_2_O without OH (ref. 45; see Supplementary Fig. 11 and Supplementary Note 7).

In addition, this island has two discriminating protrusions (the red arrows in Fig. 3a) probably corresponding to H_2_O in the second layer^7^. The structure proposed in Fig. 3e suggests that the second-layer molecules are located at the bridge site between OH and horizontal H_2_O. Because Bjerrum defects become an H-bonding acceptor for the second layer^7^, the admolecules are probably H-bonded to the lower OH group and oriented to the adjacent H_2_O.

### Capture of an H-bonging recombination

**Figure 4:**
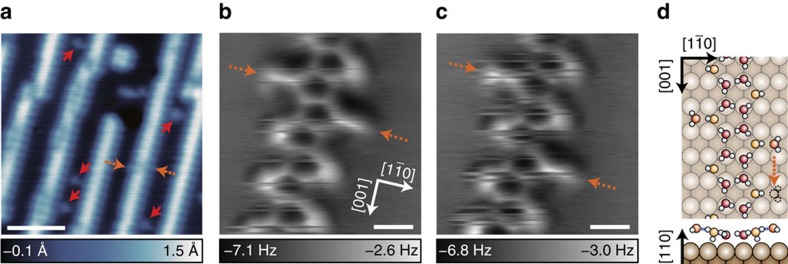
Capture of water monomers walking along a pentagonal water chain. (**a**) STM image of pentagonal H_2_O chains along with additional H_2_O monomers on Cu(110) with a CO-terminal tip (*V*=50 mV, *I*=20 pA). The solid red (dotted orange) arrows show H_2_O monomers detached from (attached to) the chains. (**b**,**c**) AFM images of two H_2_O monomers attached to the chain (*V*=0 mV, *A*=1 Å, Δ*z*=−0.5 Å). The images in **b**,**c** were obtained successively with a duration of 17 min per image. The tip height Δ*z* was set over the bare surface under the same conditions as in **a**. (**d**) Top-view (top) and side-view (bottom) schematic illustrations of a proposed atomic structure of a pentagonal chain with additional H_2_O monomers. Orange spheres represent O atoms of the attached H_2_O. Scale bars, 50 Å (**a**); 5 Å (**b**,**c**).

Finally, we demonstrate a capture of an H-bonding rearrangement. Unlike covalently bonded organic molecules, H-bonding networks can be rearranged readily. High-resolution imaging of such ‘flexible' structures allows us to trace the recombination of the bonding structure. The Cu(110) surface on which pentagonal 1D chains have been formed (Fig. 1c) was further exposed to H_2_O gas in a small amount at 6 K. The low-temperature dosing allows H_2_O molecules to adsorb onto the surface as isolated monomers which are observed as round protrusions^16^,^19^ (the solid red arrows in Fig. 4a). On the other hand, several molecules are attached to the 1D chains, which are imaged like knobs of the zigzag chain with STM (the dotted orange arrows). The AFM image in Fig. 4b shows that the additional H_2_O molecules are located at the vertices of the pentagonal rings, indicating that they are H-bonded to vertical H_2_O. During successive scanning, the additional H_2_O moved to the next vertical H_2_O (Fig. 4c). We note that the AFM images in Fig. 4b,c have scratching noises over the chains because another H_2_O molecule was attached to the tip apex together with CO and/or another H_2_O molecule (probably bonded onto horizontal H_2_O) diffused rapidly along the pentagonal chain. An interaction with the tip was probably responsible for the hopping motion of the attached molecules. As schematically shown in Fig. 4d, H_2_O monomers attached to vertical H_2_O are expected to be a horizontal configuration located on the trough between the Cu rows, which is different from the bonding site of isolated monomers (the atop site)^16^. Applying voltage pulses rarely induced the bonding of isolated monomers to the chains (Supplementary Fig. 12 and Supplementary Note 8), which is compatible with the different configurations. This result implies that vertical H_2_O in the pentagonal chains acts as an ‘active site' to trap a free H_2_O molecule. Above 150 K, the pentagonal unit with an additional H_2_O molecule may turn into a hexagonal ring (Fig. 3) via partial dissociation to yield OH groups.

## Discussion

In summary, we observe H-bonding water networks and their defects with a combination of STM and non-contact AFM. In the first layer on Cu(110), water networks are constructed by pentagonal and hexagonal H-bonding units. In the water networks, the atomic structures of local defects, which are still rendered unspecifically with STM, can be well-characterized with AFM. We also demonstrate that rearrangements of readily convertible bonds, such as H-bonds, can be traced by high-resolution AFM imaging in real time. These results reinforce that the application of AFM with molecular functionalized tips to water networks constitutes a second breakthrough—after the application of STM—in the science of water–solid interfaces.

## Methods

### Experimental setup

The STM/AFM experiments were carried out in an ultrahigh-vacuum chamber (Omicron low-temperature STM/AFM system) at 4.8 K. A tuning fork with an etched tungsten tip was used as a force sensor^47^ (resonance frequency *f*_0_=20.1 kHz, spring constant *k*_0_≈1.8 × 10^3^ N m^−1^, quality factor *Q*≈3 × 10^4^). STM images were measured in constant-current mode. AFM measurements were operated in frequency-modulation mode with an oscillation amplitude *A*=1–2 Å. The frequency shift (Δ*f*) was measured in constant-height mode at the sample bias *V*=0 mV.

Single-crystalline Cu(110) was cleaned by repeated cycles of Ar^+^ sputtering and annealing to ∼600 °C. The probe tip was sometimes poked slightly into the clean surface so that its apex was coated with Cu atoms. Distilled water was purified by freeze-and-pump cycles. The clean surface was sequentially exposed to H_2_O gas at 78 K and CO gas at 8 K via a tube doser positioned a few centimetres away from the sample surface. To achieve high-resolution images, a CO molecule coadsorbed onto the surface was picked up to attach to the tip apex^27^,^42^.

### 3D force mapping

We obtained 1024 Δ*f*(Δ*z*) curves for a pentagonal water chain on Cu(110) with a CO-terminal tip (32 point × 32 point; Fig. 1f), as described in ref. 48. Before the measurement of each curve, the tip was set over the yellow marker in Fig. 1f, and atom tracking was conducted with the tunnelling-current feedback loop closed (set point of *V*=50 mV and *I*=20 pA) in order to compensate for the thermal drift^49^. The origin of Δ*z* is defined by the tip height set at the tracking point. The positive (negative) value of Δ*z* means the tip height is further from (closer to) the sample than the set-point height. The force curve *F*(Δ*z*) at each measurement point was calculated from the Δ*f* (Δ*z*) curve by using the Sader formula^50^. The force curves are displayed after subtraction of the force curves obtained over the bare Cu surface *F*_Cu_(Δ*z*) in order to clarify the short-range force distribution (Fig. 1h). The force maps are also displayed after subtraction of the force value for the bare surface at the same Δ*z* (Fig. 1i and Supplementary Fig. 2).

### Data availability

The data that support the findings of this study are available from the corresponding author on reasonable request.

## Additional information

**How to cite this article:** Shiotari, A. & Sugimoto, Y. Ultrahigh-resolution imaging of water networks by atomic force microscopy. *Nat. Commun.* **8,** 14313 doi: 10.1038/ncomms14313 (2017).

**Publisher's note:** Springer Nature remains neutral with regard to jurisdictional claims in published maps and institutional affiliations.

## Supplementary Material

Supplementary Information Supplementary Figures 1-12, Supplementary Notes 1-8 and Supplementary References
