# Supplementary material for: Ultrahigh-resolution imaging of water networks by atomic force microscopy
Source: Nat Commun. 2017 Feb 3;8:14313. doi: 10.1038/ncomms14313 (PMC5296746; doi:10.1038/ncomms14313)
Supplement: Supplementary Information — Supplementary Figures 1-12, Supplementary Notes 1-8 and Supplementary References [file ncomms14313-s1.pdf]

Supplementary Figures

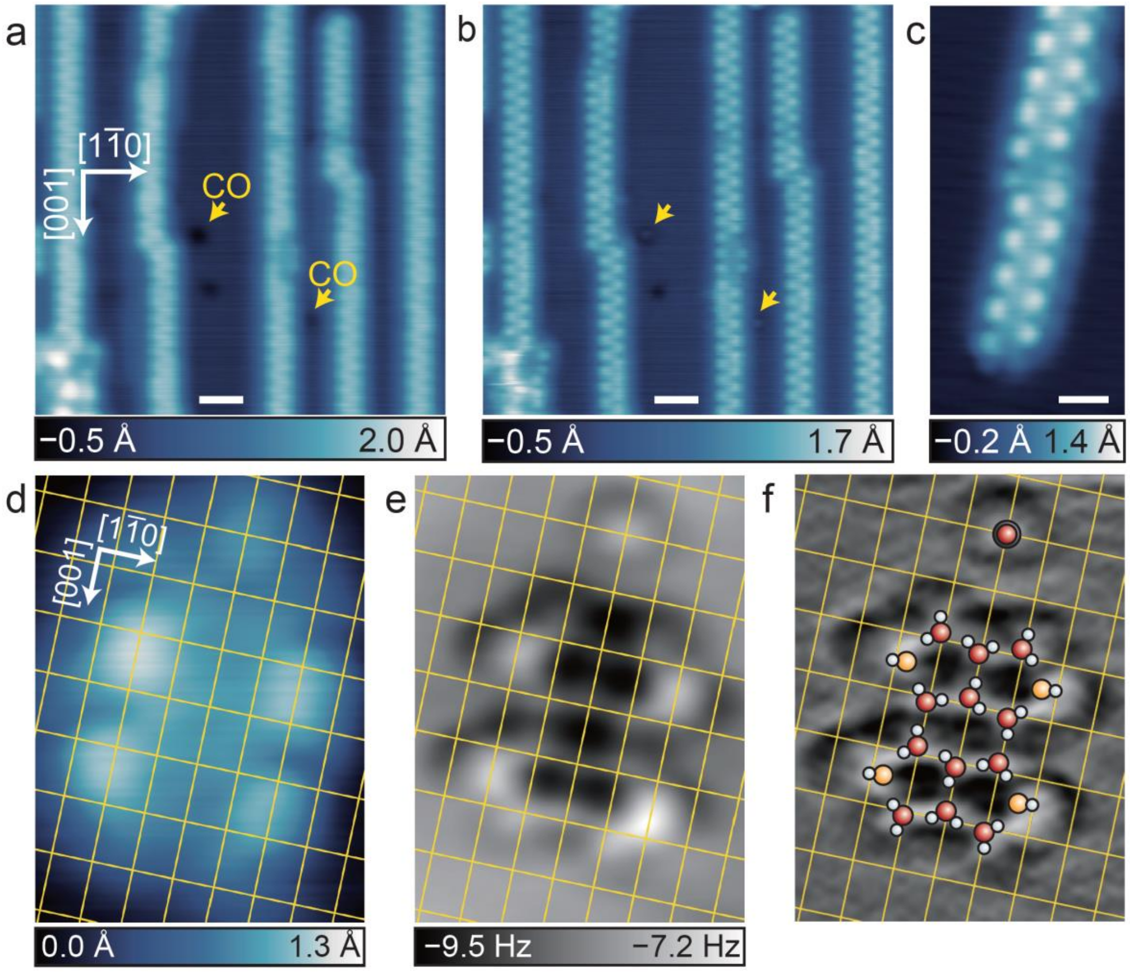

Supplementary Figure 1 | STM/AFM Observation of H_2O/Cu(110) with CO-terminal tips. a, STM image of Cu(110) after depositing water at 78 K and CO at 8 K (V = 30 mV, I = 20 pA). The image was obtained at 4.8 K with a Cu-terminal tip. **b**, STM image of the same area as in **a** but obtained with a CO-terminal tip. **c**, The same images as in Fig. 1d in the main text but without the schematic model. **d**–**f**, The same STM, AFM, and Laplacian-filtered AFM images as in Fig. 2j, k, and l in the main text, respectively, but with the yellow lines representing the lattice of surface Cu atoms (the unit cell is 2.56 Å × 3.61 Å). White, black, red, yellow spheres show H, C, O in vertical H_2O, and O in horizontal H_2O, respectively. Scale bars, 20 Å (**a**,**b**); 10 Å (**c**).

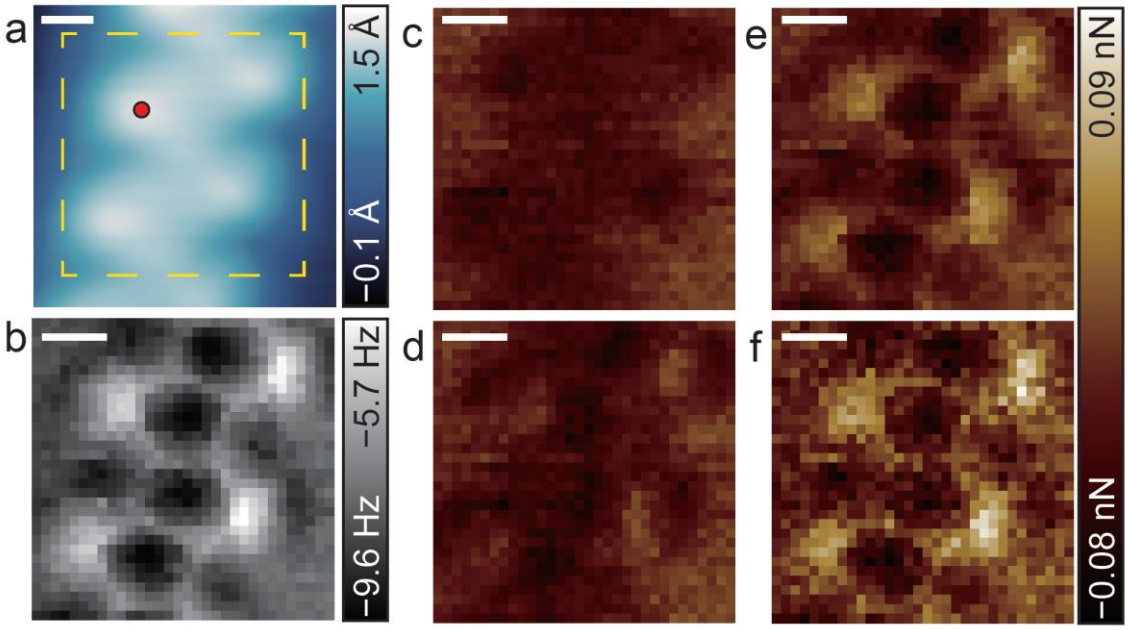

Supplementary Figure 2 | 3D mapping of the pentagonal chain. a, STM image of an intact part of the pentagonal water chain on Cu(110) (V = 50 mV, I = 20 pA). **b**, Δf map recorded within the yellow square in **a** at Δz = −2.00 Å (V = 0 V, A = 1 Å). The tip height Δz is defined as the tip height corresponding to V = 50 mV and I = 20 pA over the red marker in **a**. **c**–**f**, Short-range force maps recorded within the yellow square in **a** at Δz = −0.20 (**c**), −1.30 (**d**), −1.70 (**e**), and −2.00 Å (**f**), after subtraction of the force values for the bare Cu surface at the same Δz. Scale bar, 3 Å.

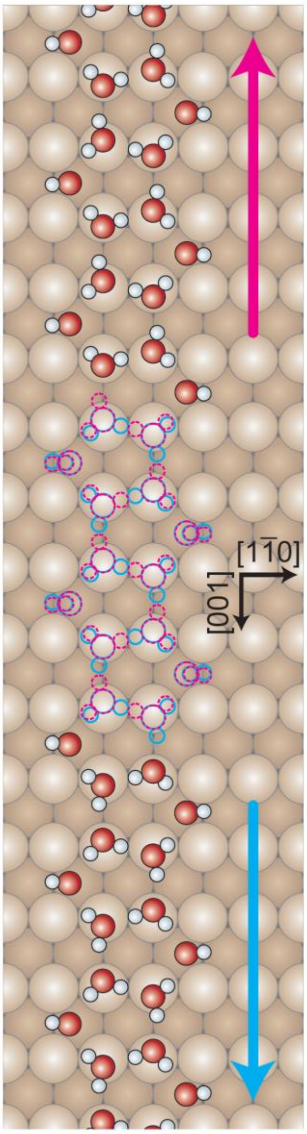

Supplementary Figure 3 | Schematic illustration of the orientation for pentagonal water chains on Cu(110). The atomic coordinates are based on ref. 1. White, red, and brown spheres represent H, O, and Cu atoms. The cyan and magenta arrows represent the opposite orientations of water chains. The cyan and magenta circles represent the atomic position of the orientations.

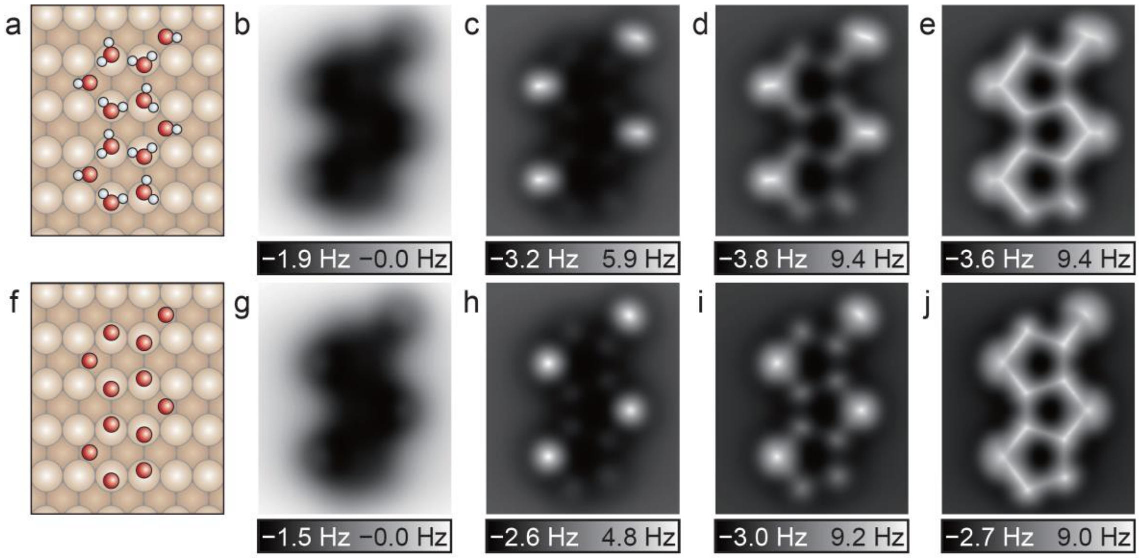

Supplementary Figure 4 | AFM simulations of the pentagonal chain on Cu(110). a, Model of the pentagonal water chain including terminals. The atomic coordinates (including Cu-atom positions) are based on ref. 1. **b**–**e**, Simulated Δf map of the model in **a** at z = 8.3 (**b**), 7.7 (**c**), 7.4 (**d**), and 7.1 Å (**e**). **f**, The same model as in **a** but with the H atoms removed. **g**–**j**, Simulated Δf map of the model in **f** at z = 8.3 (**g**), 7.7 (**h**), 7.4 (**i**), and 7.1 Å (**j**). The tip height z is defined as the distance between the top-most O atom in the models and the tip atom bonded to the flexible particle.

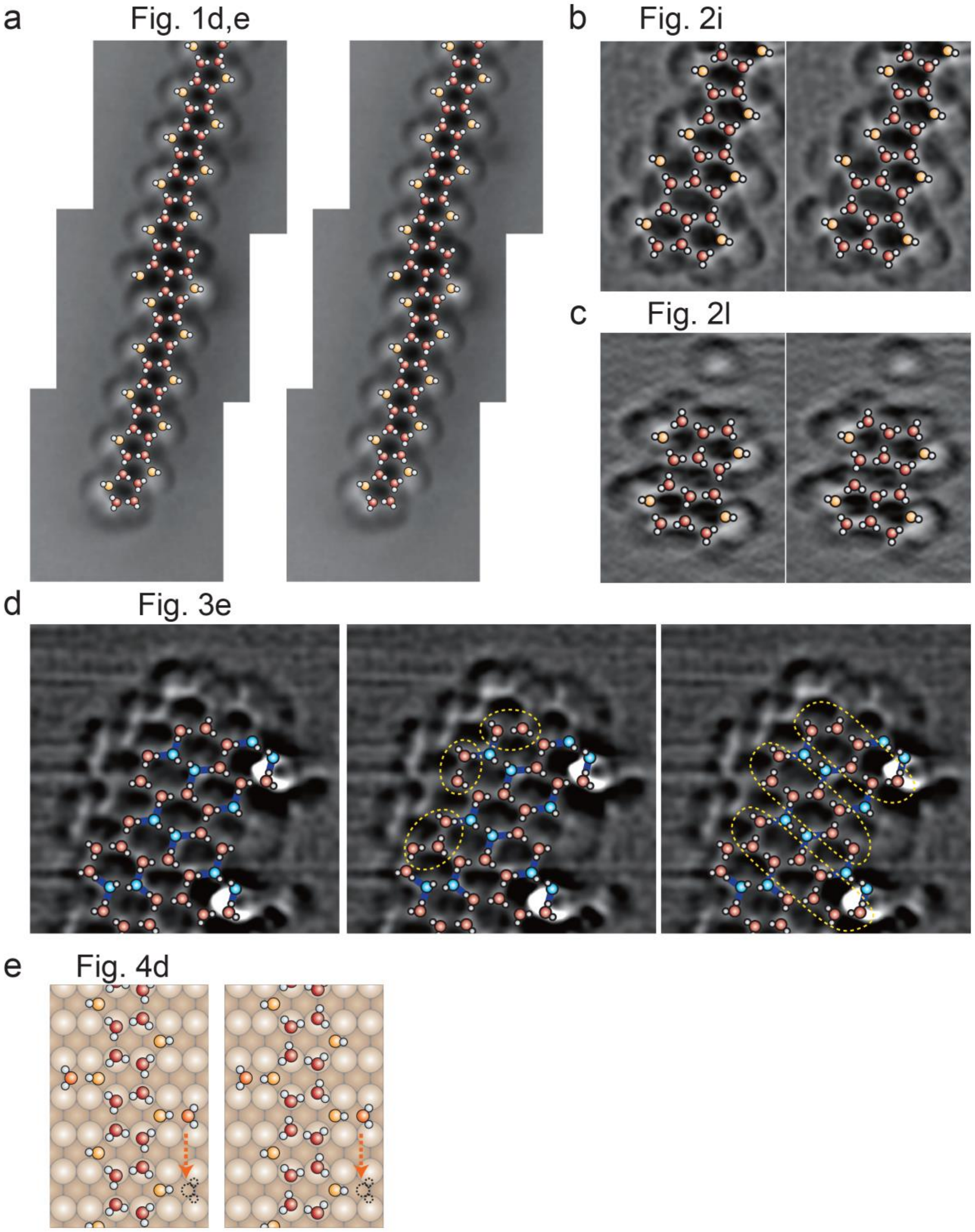

Supplementary Figure 5 | Possible atomic structures. a–**c**, All possible atomic structures of the 1D water chains shown in the main text: Fig. 1d (**a**), 2i (**b**), and 2l (**c**). **d**, Several possible atomic structures of the H_2O-OH island shown in Fig. 3e. Yellow dotted ellipses show H_2O molecules with different orientations from those in the leftmost image. **e**, All possible atomic structures of the 1D water chain with additional water monomers shown in Fig. 4d.

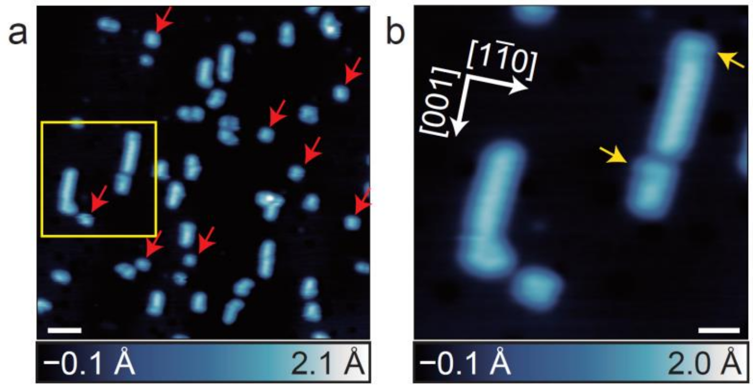

Supplementary Figure 6 | Observation of H_2O/Cu(110) at a low coverage. a, STM image of H_2O/Cu(110) at a low coverage (~0.02 monolayers). The image was acquired at 78 K with a Cu-terminal tip (V = 500 mV, I = 20 pA). The red arrows indicate the "tetraphyllous clusters." **b**, Magnified image of the region represented by the yellow square in **a**. One terminal of a short chain is imaged as a knob (the yellow arrows) whereas the other terminal has the same width as the chain. The former (latter) terminal is assigned to type iii (type i or ii), suggesting that the chains grew from a core like the "tetraphyllous cluster" to yield a terminal of type iii, and that types i and ii correspond to the "growth point" of the chain. Scale bars, 50 Å (**a**); 20 Å (**b**).

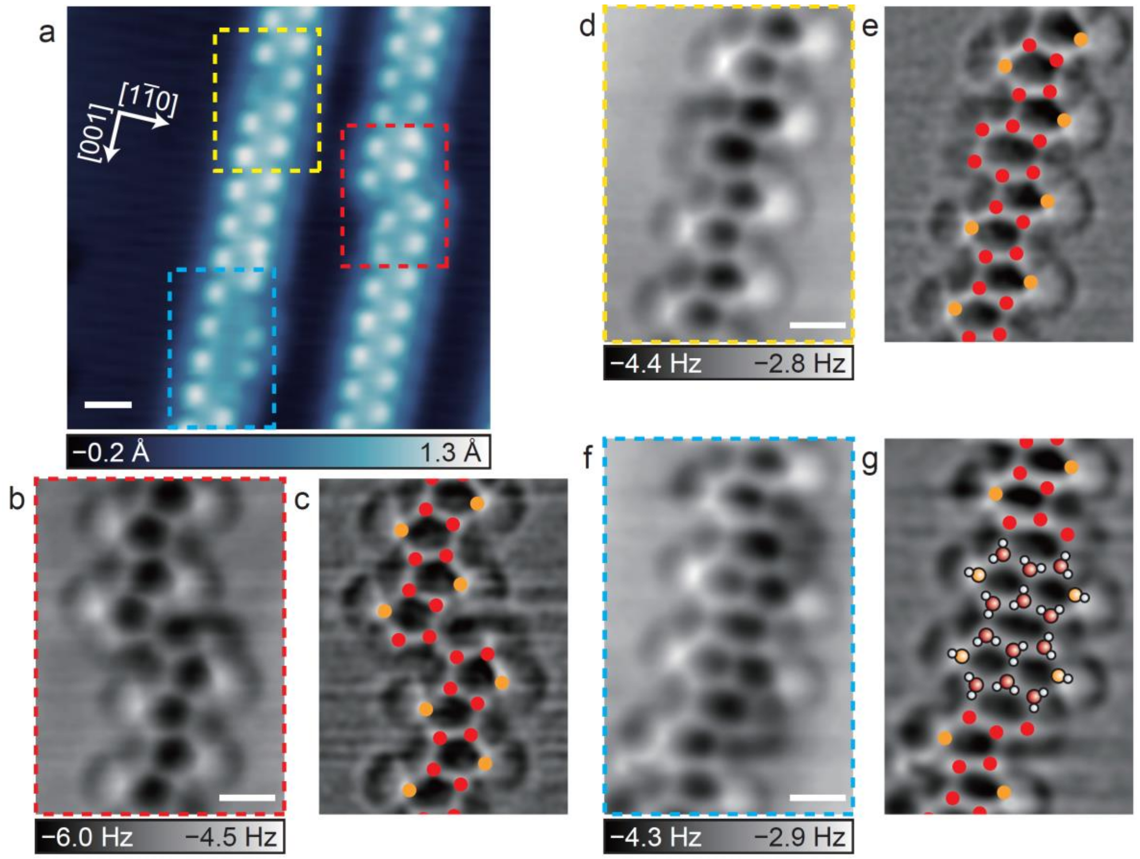

Supplementary Figure 7 | Structures of defects in the pentagonal water chains on Cu(110). a, STM images of water chains including defects with a CO-terminal tip (V = 30 mV, I = 20 pA). **b**, **d**, and **f**, AFM images of the region represented by the red, yellow, and blue squares in **a**, respectively (V = 0 mV, A = 1 Å, Δz = 0 Å). The tip height was set over the bare Cu surface under the same conditions as in **a**. **c**, **e**, and **g**, Laplace-filtered images of **b**, **d**, and **f**, respectively. Red (yellow) spheres indicate the locations of O atoms for vertical (horizontal) H_2O molecules. The atomic structure of the "tetraphyllous cluster" is also superposed in **g**. Scale bars, 10 Å (**a**); 5 Å (**b**,**d**,**f**).

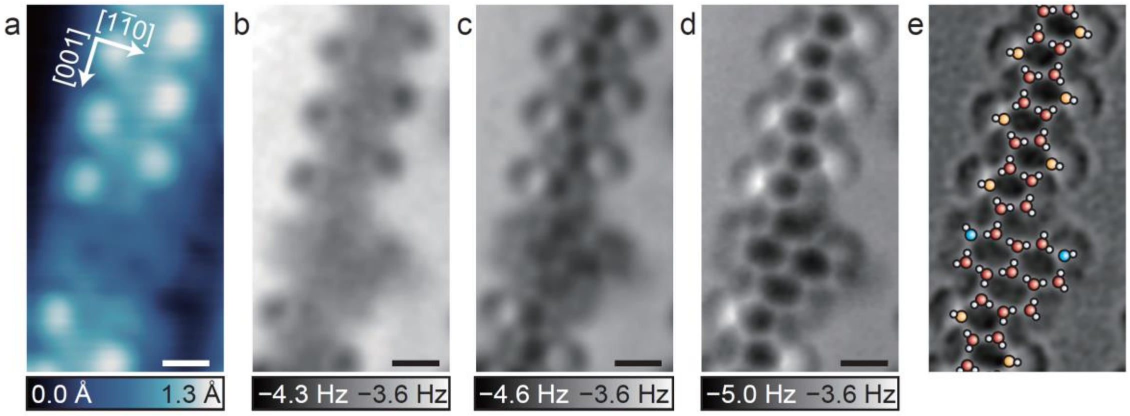

Supplementary Figure 8 | Tip-height dependence of the AFM images of the water chain. a, STM image of a water chain including a defect with a CO terminal tip (V = 30 mV, I = 20 pA). **b**–**d**, AFM images of the chain in **a** at Δz = +1.0 (**b**), +0.5 (**c**), and 0.0 Å (**d**) (V = 0 mV, A = 1 Å). The tip height was set over the bare surface under the same conditions as in **a**. **e**, Laplace-filtered image of **d**. A possible atomic structure is also shown. Red, yellow, and blue spheres represent O atoms for vertical H_2O, horizontal H_2O, and OH, respectively. Scale bar, 5 Å.

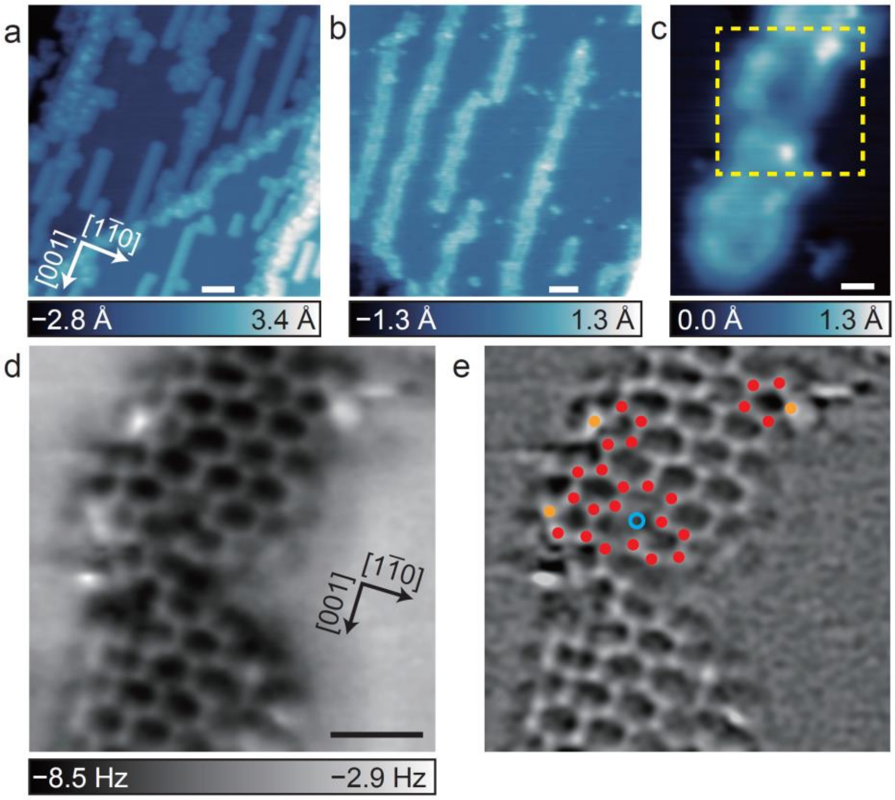

Supplementary Figure 9. a, STM image of Cu(110) after depositing water at 78 K (V = 100 mV, I = 20 pA). **b**, STM image of the same sample as in **a** but after annealing to 160 K (V = 50 mV, I = 20 pA). The images in **a** and **b** were obtained at 78 K with a Cu terminal tip. **c**, STM image of an island in the post-annealed sample, obtained at 5 K with a CO-terminal tip (V = 50 mV, I = 20 pA). **d**, AFM image of the region represented by the yellow square in **c** (V = 0 mV, A = 1 Å, Δz = −1 Å). The tip height was set over the bare surface under the same conditions as in **c**. **e**, Laplacian-filtered image of **d**. Red (yellow) spheres indicate the locations of O atoms for vertical (horizontal) H_2O molecules. A blue circle indicates a vacancy in the island. Scale bars, 50 Å (**a**,**b**); 10 Å (**c**,**d**).

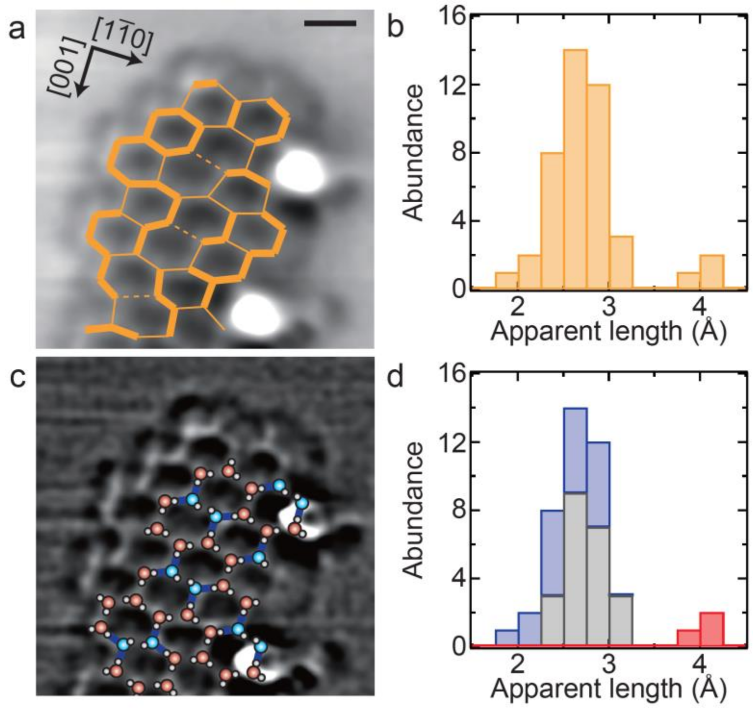

Supplementary Figure 10. **a**, AFM image of the H_2O–OH island shown in Fig. 3c in the main text. The bold solid, thin solid, and dotted lines represent the apparent O–O bonds with lengths of less than 2.7 Å, between 2.7 and 3.1 Å, and more than 3.1 Å, respectively. **b**, Histogram of the lengths of the apparent O–O bonds in **a** with the exclusion of bonds at the layer edges. **c**, Possible inside structure along with the Laplacian-filtered image of **a**. **d**, Stacking histogram of the lengths of the apparent O–O bonds for each chemical species, based on the assignment shown in **b**. The blue, grey, and red bins represent the abundances of the O–O lengths between H_2O and OH, between H_2O and H_2O, and between OH and OH, respectively. Scale bar, 5 Å.

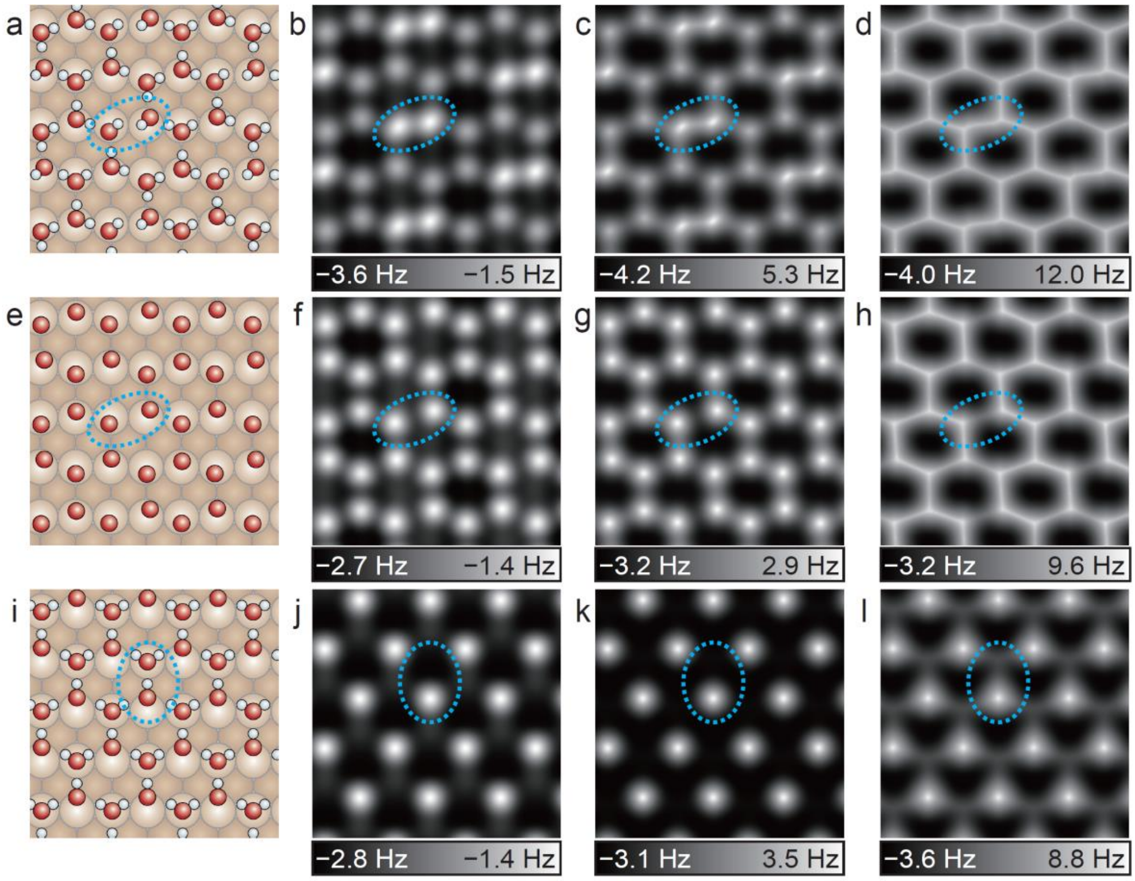

Supplementary Figure 11. a, Model of the $p(2{\times}6)$ structure consisting of a 2 H_2O : 1 OH mixture. The atomic coordinates are based on ref. 2. **b**–**d**, Simulated Δf map of the model in **a** at z = 8.1 (**b**), 7.8 (**c**), and 7.5 Å (**d**). **e**, the same model as in **a** but with the H atoms removed. **f**–**h**, Simulated Δf map of the model in **e** at z = 8.1 (**f**), 7.8 (**g**), and 7.5 Å (**h**). **i**, Model of the "H-down" structure consisting of vertical and horizontal H_2O molecules. The atomic coordinates are based on ref. 2. **j**–**l**, Simulated Δf map of the model in **i** at z = 7.3 (**j**), 7.0 (**k**), and 6.7 Å (**l**). The tip height z is defined as the distance between the top-most O atom in the models and the tip atom bonded to the flexible particle. The blue ellipses in **a**–**h** represent a Bjerrum defect. The ellipses in **i**–**l** represent the pair of a vertical H_2O and a horizontal H_2O.

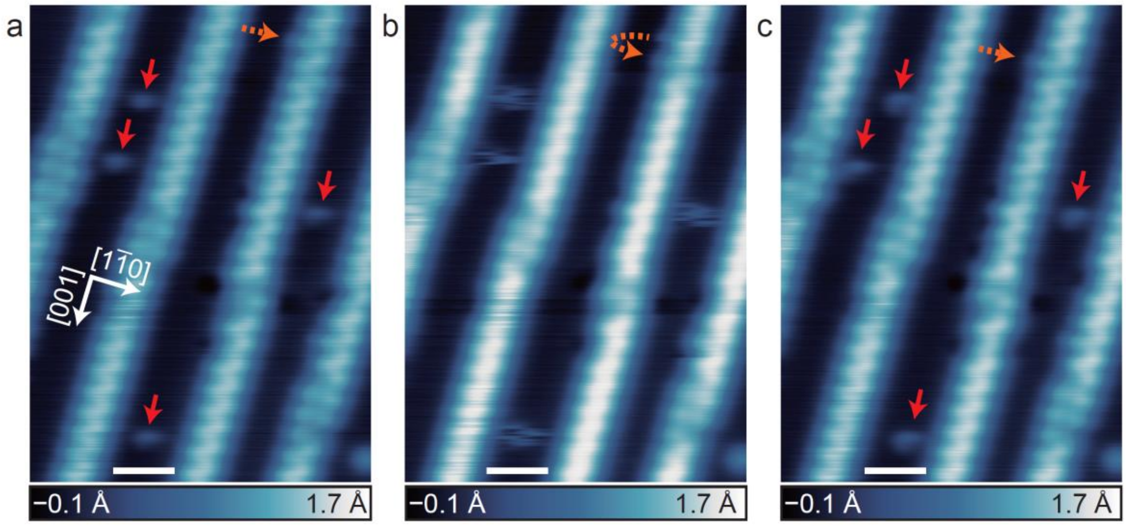

Supplementary Figure 12. a–**c**, Successively obtained STM images of pentagonal water chains with water monomers on Cu(110). The images were acquired at V = 30 (**a**), 200 (**b**), and 30 mV (**c**) and I = 20 pA with a CO terminal tip. The solid red (dotted orange) arrows show water monomers detached from (attached to) the chains. Scale bar, 10 Å.

Supplementary Notes

Supplementary Note 1: Pentagonal water chains on Cu(110)

Supplementary Fig. 1a shows a typical STM image of 1D water chains on Cu(110). For high-resolution STM/AFM images, a CO molecule was picked up from the surface to attach to the tip apex[3,4]. CO molecules on the surface are observed as round depressions by STM with a Cu-terminal tip (the yellow arrows in Supplementary Fig. 1a), whereas the STM images of CO turn into protrusions after picking up a CO molecule (the yellow arrows in Supplementary Fig. 1b). The zigzag shape of the STM image for the water chains is emphasized by using the CO-terminal tip (Supplementary Fig. 1b,c).

Coadsorbed CO molecules are also helpful to determine the adsorption sites of water molecules. Supplementary Fig. 1d,e shows STM and AFM images, respectively, of the "tetraphyllows cluster" along with a CO molecule (see Fig. 2j–l). On Cu(110), CO is bonded to the atop site with an upright configuration[5,6]. The grid lines in the figures represent the lattice of Cu atoms on the surface, based on the location of the CO molecule. Therefore, the adsorption sites of the water molecules in the cluster are determined as shown in Supplementary Fig. 1f. O atoms of the horizontal (vertical) H_2O are located near the atop (hollow) sites, in good agreement with the calculated structure of the 1D water chain on the surface[1].

Supplementary Note 2: Force maps of the pentagonal chain

As shown in Fig. 1f–i in the main text, we obtained the 3D mapping data for a pentagonal water chain. The measurement region is represented by the yellow square in Supplementary Fig. 2a. The Δz dependence of the force maps is shown in Supplementary Fig. 2c–f. The force maps are displayed after subtraction of the force value obtained over the bare Cu surface at the same Δz in order to clarify the short-range force distribution (see Methods in the main text). At a high Δz, vertical H_2O is imaged as a round depression (Supplementary Fig. 2c). Because vertical H_2O is more protruded than horizontal H_2O (see Fig. 1b), vertical H_2O yields a larger attractive interaction with the tip at the tip height. With decreasing tip height, the Pauli repulsion between vertical H_2O and the tip becomes dominant whereas horizontal H_2O is still attractive (Supplementary Fig. 2d). At $\Delta z \leq -1.70$ Å, interaction forces over both the vertical and horizontal H_2O molecules are repulsive, giving rise to oxygen-skeleton images (Supplementary Fig. 2e,f). The shape of

the force map in Supplementary Fig. 2f is similar to that of the *Δf* maps at the tip height (Supplementary Fig. 2b). Note that dissipation signals even at the closest tip height (Δz = −2.00 Å) were below the noise level (~2 meV).

Supplementary Note 3: AFM simulations of the pentagonal chain

Supplementary Fig. 3 shows the top-view schematic of the pentagonal water chain on Cu(110). The atomic coordination of the pentagonal structure was provided by Carrasco *et al.* (ref. 1). The periodic interval of the zigzag chain is 7.2 Å along the [001] direction. A pentagonal ring of the chain consists of one horizontal H_2O and four vertical H_2O. Horizontal H_2O is H-bonded to one vertical H_2O and another horizontal H_2O as an H donor and to another horizontal H_2O as an acceptor. On the other hand, vertical H_2O has two non-H-bonded H atoms and works as an acceptor for two horizontal H_2O molecules. In Supplementary Fig. 3, the chain represented by the magenta arrow corresponds to the 180°-rotated chain represented by the cyan arrow. The atomic positions in these chains cannot be overlapped by parallel displacement, resulting in the directionality of the chain. However, the positions of the oxygen atoms are almost coincident between these orientations (see the centre part in Supplementary Fig. 3), which is responsible for the symmetric pentagonal rings in the AFM image in Fig. 1e in the main text.

To confirm the contribution of H atoms to the AFM images, we conducted AFM simulations using the Probe Particle Model provided by Hapala *et al.* (refs 7–10). The surface Cu atoms were also considered in the simulations. Here the electrostatic field of the sample[8] was excluded. It is noteworthy that, in the simulations, the atomic positions of the sample were fixed (i.e., the relaxation of the adsorbate molecules was not considered).

Supplementary Fig. 4a shows the structural model we used. The bottom (upper) part of the chain is comparable to a terminal of type i (type ii) as shown in Fig. 2a–c (2d–f) in the main text. Supplementary Fig. 4b–e shows the tip-height dependence of the simulated *Δf* images. The appearances of the images are quite similar to the experimental force maps in Supplementary Fig. 2c–f. In particular, the oxygen-skeleton images are successfully reproduced in Supplementary Fig. 4d,e. Remarkably, H atoms are not visualized clearly in the simulated images. To confirm that, we use another model in which all H atoms are removed (Supplementary Fig. 4f). The simulated AFM images of this model

(Supplementary Fig. 4g–j) are quite similar to the original images (Supplementary Fig. 4b–e). To detect the H atoms with AFM, *Δf* imaging with tips functionalized by another molecule/atom[9, 11], Kelvin probe force microscopy[12], and induction of H-atom transfer in the network[13, 14] would be helpful.

Supplementary Note 4: Possible atomic structures

Supplementary Figure 5a–c shows all possible structures of the water chains and cluster shown in the main text. The horizontal H_2O molecules in the hexagonal rings can have two kinds of orientations.

Supplementary Fig. 5d shows several possible structures of the H_2O-OH island (Fig. 3e). Because the chemical species and configurations of the island edges are unknown, several orientations of H_2O molecules are conceivable. However, in all of the possible combinations, the atomic structures and locations of OH groups (i.e., Bjerrum defects) are unchanged.

Supplementary Fig. 5e shows all possible structures of the 1D water chain with additional water monomers (Fig. 4d). Because the direction of H bonds (i.e., the locations of H atoms) in the water chain cannot be discriminated, two kinds of atomic structures are conceivable.

Supplementary Note 5: Other kinds of defects in pentagonal water chains

We observed several kinds of defects in the water chains. Supplementary Fig. 7a shows STM images of a kink (represented by the red rectangle) and defects (the yellow and blue rectangles). With AFM, the kink is imaged as single-bonded pentagonal rings (Supplementary Fig. 7b,c). This structure is presumed to originate from an accidental collision of two type-i terminals (see Fig. 2a–c) to yield a single H-bond between the pentagonal rings. As shown in Supplementary Fig. 7d,e, the defect represented by the yellow rectangle in Supplementary Fig. 7a is ascribed to fused hexagonal rings, substituting for a pentagonal unit. This structure is analogous to the kink with a hexagonal unit shown in Fig. 1d,e. The defect represented by the blue rectangle in Supplementary Fig. 7a has a more complex structure; here, hexagonal rings and twin pentagonal rings are arranged alternately (Supplementary Fig. 7f). This network includes the structure of a “tetraphyllous cluster” (Supplementary Fig. 7g), suggesting two opposite chains grew

from the core structure.

STM and AFM images of another characteristic defect are shown in Supplementary Fig. 8a and d, respectively. The AFM image indicates that the defect consists of three fused hexagonal rings. It is noteworthy that the H-bonding arrangement of the fused hexagonal rings cannot be built only by horizontal H_2O molecules. Thus some OH groups must be included in the structure, as shown schematically in the example in Supplementary Fig. 8e. However, we cannot identify the specific sites of the OH groups because the Δf intensity at each vertex of the hexagons is almost identical at several tip heights (Supplementary Fig. 8b–d). This supports the idea that AFM does not image H-bonds themselves but predominantly reflects the O-atom positions. Such defects were probably formed by the existence of dissociative products of water and/or impurities. Although water dissociation on the surface occurs above 150 K (ref. 15), the reaction may proceed even at 78 K in extremely low yield.

In addition, the upper parts in Supplementary Fig. 8b–d show the tip-height dependence of Δf images for an intact pentagonal chain. The Δz dependence of the appearances are quite similar to the force maps (Supplementary Fig. 2c–f) and the simulated Δf images (Supplementary Fig. 4b–e).

Supplementary Note 6: Hexagonal water–hydroxyl islands on Cu(110)

The H_2O–OH island in Fig. 3 in the main text was prepared by the following procedure. A clean Cu(110) surface was exposed to H_2O gas at 78 K, so that pentagonal water chains were formed on the surface (Supplementary Fig. 9a). After that, the sample was annealed to 160 K for 15 min, followed by re-cooling to 78 K and measured with STM. Then protruded bands were observed (Supplementary Fig. 9b). The bands almost follow along the [001] direction but are slightly crooked. This structure is quite similar to the appearance of H_2O/O/Cu(110) at 155 K (ref. 16).

We observed another island with AFM at 4.8 K. Although the CO-terminal tip which we used for Fig. 3 allows the honeycomb structure to be observed with STM, the inside structure is invisible in an STM image with another CO-terminal tip (Supplementary Fig. 9c). Even with the tip, the oxygen skeleton is visualized with AFM (Supplementary Fig. 9d). In this island, several pentagonal rings are located at the edge (Supplementary Fig. 9e), suggesting that the pentagonal units, which are a dominant

structure at 78 K, partially remain. Furthermore, the blue circle in Supplementary Fig. 9e indicates a vacancy within the hexagonal network. These local structures are hardly identified by the STM image (Supplementary Fig. 9c), further evidencing the tremendous advantage of the AFM measurements.

Next, we consider the details of the assignment for the hexagonal network in Fig. 3c (shown again in Supplementary Fig. 10c). The superposed lines in Supplementary Fig. 10a show the apparent O–O bonds in the oxygen skeleton of the island. The line shapes represent the length d of the apparent O–O bonds: $d < 2.7$ Å (bold solid line), 2.7 Å $\leq d \leq$ 3.1 Å (thin solid line), and $d > 3.1$ Å (dotted line). As shown by the histogram of d (Supplementary Fig. 10b), the dotted bonds are much longer than the other bonds, and therefore, they are assigned to Bjerrum defects (i.e., OH–OH pairs without H bonds between them).

Based on the arrangement of the $p(2\times6)$ structure[2, 17], the inside structure of the islands is assigned in a reasonable manner as shown in Supplementary Fig. 10c. On the other hand, the edge bonds have relatively short O–O lengths (the bold solid lines at the island edges in Supplementary Fig. 10a), suggesting that the edges have different structures from the intact $p(2\times6)$ network in order to stabilize the abortive H-bonding network. Therefore, the edges were not considered here. To confirm the validity of the assignment, we show the relationship between the chemical species and the apparent lengths as a stacking histogram in Supplementary Fig. 10d. The bins are color-coded according to the assigned species: H_2O–OH (blue), H_2O–H_2O (grey), and OH–OH (red). The histogram indicates that the distance between H_2O molecules is basically longer than that between H_2O and OH, in agreement with the property of H bonds; OH acts as a stronger H accepter than H_2O and thus yields a stronger H bond (the blue lines in Supplementary Fig. 10c). The apparent length between OH and OH (4.0 ± 0.2 Å) is much longer than that in the $p(2\times6)$ model[2] (3.2 Å). This is probably because the finite network in the small islands allows the atomic positions to be easily modified. In AFM images with CO-terminal tips, the apparent bond lengths are magnified from the actual bond length due to the relaxation of CO (refs 9, 18), which also contributes to the difference of the lengths.

Supplementary Note 7: AFM simulations of the hexagonal network

We also conducted AFM simulations for the hexagonal network shown in Fig. 3,

using the Probe Particle Model provided by Hapala *et al.* (refs 7–10) in the same manner as described in Supplementary Fig. 4. Supplementary Fig. 11b–d shows simulated AFM images of the $p(2\times6)$ structure with Bjerrum defects[2, 17] (Supplementary Fig. 11a). At a shorter tip height, the image appears as a honeycomb network where all of the oxygen sites have almost the same intensity (Supplementary Fig. 11d) because the image predominantly reflects the repulsive interaction from the O atoms. In Supplementary Fig. 11b,c, on the other hand, the Bjerrum defects (the blue ellipse in Supplementary Fig. 11a) are slightly more protruded than the other oxygen sites. In the model, the H atoms of OH groups are the most protruded, whereas the geometric heights of all O atoms are almost the same[2]. Therefore, the protrusions at the defects originate from the top-most H atoms. These results disagree with the experimental images, in which the OH groups are not protruded (see Fig. 3b). To investigate the disparity, we simulated another model in which H atoms are removed from the $p(2\times6)$ arrangement (Supplementary Fig. 11e). In this model, the intensities of each oxygen seemed almost the same, in good agreement with the experimental images. The apparent bonds at Bjerrum defects (i.e., bonds with the longest O–O distance) have slightly weaker intensities than the other bonds (Supplementary Fig. 11f–h), giving rise to a similar appearance as in Fig. 3c. We assume that the top-most H atoms of OH groups are relaxed and tilted by the approaching tip, accounting for the absence of a significant effect of the H atoms on the AFM images.

We also simulated another hexagonal network model of the "H-down" structure[2] (Supplementary Fig. 11i). This network consists of a pair of a vertical H_2O and horizontal H_2O (the blue ellipse in Supplementary Fig. 11i). The vertical H_2O molecules, which are located near the Cu atomic row, are ~0.5 Å more protruded than the horizontal H_2O. Owing to the atomic corrugations, the simulated AFM images do not appear as hexagonal rings (Supplementary Fig. 11j–l), and these images are very different in appearance from their experimental counterparts.

Supplementary Note 8: Pentagonal water chains with additional water monomers

Water molecules form pentagonal chains on Cu(110) at 78–140 K (refs 1, 19), whereas the molecules are adsorbed as monomers under the extremely low coverage at ~6 K (refs 14, 20). Figure 12a shows an STM image of the same sample as Fig. 4 in the main text. The additional H_2O molecules are mainly observed as isolated monomers (the solid red arrows). Sample biases of more than ~40 mV induce isolated monomers on the

surface to diffuse along the atomic Cu row (the $[1\bar{1}0]$ direction)[20]. At $V = 200$ mV, the additional H_2O molecules in Supplementary Fig. 12a diffuse along the Cu row, but the hopping motion is restricted by the chains (Supplementary Fig. 12b). Even after the diffusion, the isolated monomers are not attached to the chains (Supplementary Fig. 12c), suggesting a repulsive interaction between the isolated monomers and the chains.

On the other hand, a few water molecules are attached to the water chain (the dotted orange arrows in Supplementary Fig. 12a). At $V = 200$ mV (Supplementary Fig. 12b), an attached molecule moved to the next site (near vertical H_2O) along the [001] direction (Supplementary Fig. 12c), in analogy with the hopping motion observed by AFM (see Fig. 4b,c). The difference of the hopping direction between the isolated and attached monomers implies that the adsorption structures are different.

Supplementary References

1. Carrasco, J. *et al.* A one-dimensional ice structure built from pentagons. *Nat. Mater.* **8**, 427–431 (2009).
2. Baghbanpourasl, A., Hingerl, K., Wippermann, S. & Schmidt, W. G. Copper(110) surface in thermodynamic equilibrium with water vapor studied from first principles. *Surf. Sci.* **612**, 82–89 (2013).
3. Bartels, L., Meyer, G. & Rieder, K.-H. Controlled vertical manipulation of single CO molecules with the scanning tunneling microscope: A route to chemical contrast. *Appl. Phys. Lett.* **71**, 213–215 (1997).
4. Gross, L., Mohn, F., Moll, N., Liljeroth, P. & Meyer, G. The chemical structure of a molecule resolved by atomic force microscopy. *Science* **325**, 1110–1114 (2009).
5. Hofmann, P., Schindler, K. M., Bao, S., Fritzsche, V., Bradshaw, A. M. & Woodruff, D. P. A photoelectron diffraction study of the structure of the Cu{110}(2×1)-CO system. *Surf. Sci.* **337**, 169–176(1995).
6. Lorente, N. & Ueba, H. CO dynamics induced by tunneling electrons: differences on Cu(110) and Ag(110). *Eur. Phys. J. D* **35**, 341–348 (2005).
7. Hapala, P. *et al.* Mechanism of high-resolution STM/AFM imaging with functionalized tips. *Phys. Rev. B* **90**, 085421 (2014).
8. Hapala, P., Temirov, R., Tautz, F. S., & Jelínek, P. Origin of high-resolution IETS-STM images of organic molecules with functionalized tips. *Phys. Rev. Lett.* **113** 226101 (2014).
9. Hapala, P. *et al.* Mapping the electrostatic force field of single molecules from high-resolution scanning probe images. *Nat. Commun.* **7**, 11560 (2015).
10. Hapala, P., Probe Particle Model. Available: https://github.com/ProkopHapala/ProbeParticleModel [Accessed: 15 Aug. 2016].
11. Mohn, F., Schuler, B., Gross, L., & Meyer, G. Different tips for high-resolution atomic force microscopy and scanning tunneling microscopy of single molecules. *Appl. Phys. Lett.* **102**, 073109 (2013).
12. Mohn, F., Gross, L., Moll, N., & Meyer, G. Imaging the charge distribution within a single molecule. *Nat. Nanotechnol.* **7**, 227–231 (2012).

13. Kumagai, T., Shiotari, A., Okuyama, H., Hatta, S., Aruga, T., Hamada, I., Frederiksen, T, & Ueba, H. H-atom relay reactions in real space. *Nat. Mater.*, **11**, 167–172 (2012).

14. Meng, X., Guo, J., Peng, J., Chen, J., Wang, Z., Shi, J. R., Li, X. Z., Wang, E. G. & Jiang, Y. Direct visualization of concerted proton tunnelling in a water nanocluster. *Nat. Phys.* **11**, 235–239 (2015).

15. Andersson, K. *et al.* Molecularly intact and dissociative adsorption of water on clean Cu (110): A comparison with the water/Ru (001) system. *Surf. Sci.* **585**, L183–L189 (2005).

16. Pang, Z. *et al.* Adsorption and reactions of water on oxygen-precovered Cu(110). *J. Phys. Chem. C* **120**, 9218–9222 (2016).

17. Forster, M., Raval, R., Hodgson, A., Carrasco, J. & Michaelides, A. *c*(2×2) water-hydroxyl layer on Cu(110): a wetting layer stabilized by Bjerrum defects. *Phys. Rev. Lett.* **106**, 046103 (2011).

18. Gross, L. *et al.* Bond-order discrimination by atomic force microscopy. *Science* **337**, 1326–1329 (2012).

19. Yamada, T., Tamamori, S., Okuyama, H. & Aruga, T. Anisotropic water chain growth on Cu(110) observed with scanning tunneling microscopy. *Phys. Rev. Lett.* **96**, 036105 (2006).

20. Kumagai, T. *et al.* Water Monomer and Dimer on Cu (110) Studied Using a Scanning Tunneling Microscope. *e-J. Surf. Sci. Nanotechnol.* **6**, 296–300 (2008).
